
JOURNAL INFORMATION
==============================
NLM Title Abbreviation: ScientificWorldJournal
Journal ID: ScientificWorldJournal
NLM Journal ID: 101131163
Journal ID: TSWJ

The Scientific World Journal

ISSN: 2356-6140
EISSN: 1537-744X
Publisher: Wiley

ARTICLE INFORMATION
==============================
PMCID: PMC6083964
DOI: 10.1100/tsw.2001.430
Article ID: 161379
Article version: 1
Subjects: Extended Abstract

CGRP as a Migraine Inducing Substance

Olesen J.
Lassen L. H.
Department of Neurology, Glostrup Hospital, University of Copenhagen and Copenhagen Headache Center, DK-2600 Glostrup, Copenhagen, Denmark
Publication date: 2001
Electronic publication date: 2001 Dec 8
Volume: 1
First page: 31
Last page: 31
Copyright: Copyright © 2001 J. Olesen and L. H. Lassen.
Copyright year: 2001
License: This is an open access article distributed under the Creative Commons Attribution License, which permits unrestricted use, distribution, and reproduction in any medium, provided the original work is properly cited.
License URL: https://creativecommons.org/licenses/by/3.0/

==============================
4th International Meeting on Calcitonin Gene-Related Peptide (CGRP)
*TheScientificWorld* (2001) 1(S1), 31
ISSN 1532-2246; DOI 10.1100/tsw.2001.430

### CGRP AS A MIGRAINE INDUCING SUBSTANCE

J. Olesen and L.H. Lassen
Department of Neurology, Glostrup Hospital, University of Copenhagen and Copenhagen Headache Center, DK-2600 Glostrup, Copenhagen, Denmark

Calcitonin gene-related peptide (CGRP) has been detected in increased amounts in external jugular venous blood during migraine attacks. However, it is unknown whether this is secondary to migraine or whether CGRP may cause headache.

In a double-blind, crossover study the effect of human αCGRP (2 μg/min) or placebo infused i.v for 20 min was studied in 12 patients suffering from migraine without aura. Headache intensity was scored on a scale from 0 to 10.

Two patients were excluded due to severe hypotension and one because she had an infection. In the first hour median peak headache score was 1.0 in the hαCGRP group vs. 0 in the placebo group ($p < 0.01$). In the following 11 h all patients experienced headache after hαCGRP versus one patient after placebo ($p = 0.0004$). The median maximal headache score was 4 after CGRP and 0 after placebo ($p = 0.006$). In three patients after hαCGRP but in no patients after placebo did the delayed headache fulfill the IHS criteria for migraine without aura. Since i.v. administration of hαCGRP causes headache and migraine in migraineurs, our study suggests that the increase in CGRP observed during spontaneous migraine attacks may play a causative role.
